# Analysis of Hospital Costs in Road Traffic Injuries

**DOI:** 10.30476/BEAT.2021.86855

**Published:** 2021-01

**Authors:** Sara Emamgholipour, Mehdi Raadabadi, Mohammadhosein Dehghani, Saeed Fallah-Aliabadi

**Affiliations:** 1 *Department of Health Management and Economics, School of Public Health, Tehran University of Medical Sciences, Tehran, Iran*; 2 *Trauma Research Center, Shahid Sadoughi University of Medical Sciences, Yazd, Iran*; 3 *Department of health in emergencies and disaster, School of public Health, Shahid Sadoughi University of medical Sciences, Yazd, Iran*

**Keywords:** Road traffic injuries (RTI), Hospital costs, Length of stay (LOS), Injury

## Abstract

**Objective:**

To investigate the factors affecting the hospital costs in the road traffic injuries.

**Methods:**

This applied study examined the information of patients presenting to Yazd Trauma Center in 2018. The data were extracted from Comprehensive Traffic Injuries System affiliated to the center, which were described with frequency, percentage, mean, and SD, and then analyzed using independent t-test and one-way ANOVA.

**Results:**

Most injuries (%66.4) are caused by motorcycle and pertained to head region (%61.8). Some significant correlations were found among gender, type of injury, patient’s final status, site of road accident, patient’s nationality, type of vehicle used at the time of accident, length of stay (hospital stay), patient’s age, and hospital costs (*p*<0.05). Moreover, the costs were higher in men, and in those with head and neck injuries, dead casualties, suburban high-way accidents, motor cyclists, hospital stay longer than three days, and older patients.

**Conclusion:**

Given the significant correlations between demographic and social variables under study, the results may be used in planning and designing strategies for controlling road traffic injuries and reducing the related hospitalization costs.

## Introduction

Around 1.2 million road traffic deaths and between 20 and 50 million non-fatal Road Traffic Injuries (RTIs) annually occur worldwide [1], making them the ninth leading cause of death [2]

In 2015, 1.5 million people around the world lost their lives due to transportation trauma [3]. It is also predicted that, with increasing traffic accidents, this rate will increase up to 8 million deaths per year until 2020 [4]. 

According to the published statistics, the load of traffic casualties and deaths is heavier in East Mediterranean (EMR) and North Africa compared to other regions of the world. In addition, the EMR region has the highest traffic mortality rate [5]. Among countries, Afghanistan, Oman, and Iran have the highest rate of road traffic injuries (RTI). Iran had the highest mortality rate among middle-income countries [6]. According to DALY Statistics in 1990, RTIs have exerted severe effects on Iran and the neighboring Arab countries, including Bahrain, Saudi Arabia, Kuwait, Oman, Qatar, and UAE, which consequently imposed significant costs on these countries in humanistic, social, and economical domains [7-9]. Considering lifespan, due to premature death, traffic accidents ranked as the second after cardiovascular diseases during 2005-2015 [3].

These injuries and deaths have great social and economic impacts by creating a significant financial burden for individuals and families, as well as for the health care system [10], so that about %3 of global gross production is annually devoted to traffic accidents [1]. Regarding the economic consequences of RTIs such as the lost revenue, humanistic costs, and healthcare services’ costs, RTIs receive approximately %1 of national gross production in low-income countries, %1.5 in average-income countries, and %2 in high-income countries [11]. 

Annual RTI costs in developing countries like Iran is %6.64 of national gross production (39 billion US dollars) [9]. Another study estimated annual RTI costs in Iran as 2 billion US dollars (about %2 of national gross production (NGP)) [12]. However, if the present trend of RTIs continues with no efficient interventions, RTIs will be the fifth leading cause of mortality and morbidity by 2030 worldwide [13]. Consequently, considering the heavy traffic loads and the increased hospital costs, having more accurate knowledge of the economic effects of traffic accidents is mandatory. At the policy-making level, the results of this study are urgently needed in supervising the efficacy and efficiency of traffic safety policies and also in revising the new policies. Moreover, our findings concerning the correlation among the type of application of roads, type of damage and probable hospitalization, and hospitalization costs may predispose the improvement of the present transportation models and thus resulting in the optimization of assessment of new policies. Additionally, information on damages with the highest rates of costs ought to urge the transportation industry to both recognize and control different types of approaches related to these damages and injuries. This information can be subsequently used in developing innovative traffic safety technologies. Additionally, these findings are useful at the small scale for hospital managers of trauma centers in developing economical and clinical programs [14]. 

Regarding traffic accidents, although some studies have been conducted on the prevalence, type of injuries, and severity of accidents, few studies have investigated the correlation between hospital costs of traffic casualties and variables affecting them [15, 16]. Furthermore, this study has classified the sources of extraction of injury types in terms of ICD standard and thus, provided a better comparability for researchers and scholars from other countries. Considering the above-mentioned issues, this study explored the factors affecting hospital costs of traffic accident casualties. 

## Materials and Methods

This applied study was performed in 2019. In addition, this research used a cross-sectional design to examine information on traffic accidents in Yazd Trauma Center in 2018. 

This center, located in the north-to-south route of the country, is known as the pole of trauma in the center and south-east of the country. Therefore, according to Iranian Insurance Company statistics, %30-40 of patients and casualties of this center come from the neighboring provinces. Besides, this trauma center is located in a province that, proportional to its population, has high rates of traffic and motor accidents, and the traffic accident mortality prevalence is reported to be 46 deaths per 100,000 cases [17]. 

The required information was extracted from RTI System affiliated to this center. Previous studies obtained their information from some sources such as road traffic accidents, Legal Medicine Organization, traffic police statistics, and hospital sources. However, these valuable data seemed to be improper for performing epidemiological and statistical analyses to determine causes of road traffic accidents and to develop preventive intervention programs, may be due to inaccurate definitions of some variables and lack of registration of some necessary information in these forms such as patient’s costs, hospital indices, and multi-source information. Another weakness of the previous information sources was lack of diagnosis of accident-related disease in terms of the International Classification of Disease (ICD). Hence, this study used the comprehensive RTI registration system in which the diagnosis and type of trauma are considered based on ICD. The information extracted from this system were as follows: time of admission, time of discharge, the patient’s age, the patient’s gender, total cost of services, type of injury, the patient’s final status, day and hour of the accident occurrence, location of accident, and the patient’s nationality. Some information such as educational level and the patient’s occupation were excluded, since they were deficient. The gleaned data were coded and then imported to SPSS. Finally, the data were described with frequency, percentage, mean, and SD, and analyzed using independent t-test and one-way ANOVA. Excel was used to plot the curves. 

## Results

Totally, the information of 8105 RTI patients were investigated during the time interval of the study. Most of the patients (%75.7) were men and aged between 16 and 30 years old (%42.1). In addition, most injuries caused by motor cycle (%66.4) occurred in the head region (%61.8). The mean hospital cost was 20.02 million Rials. The mean hospital cost was statistically significant among gender groups, type of injury, the patient’s final status, the patient’s nationality, and type of vehicle at the time of accident (Tables 1 and 2).

**Fig. 1:**
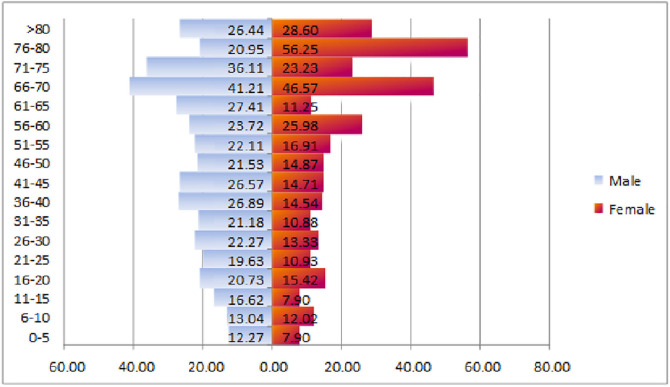
Hospital costs’ distribution pyramid in terms of age groups and gender

Distribution of hospital costs in terms of age groups indicated that hospital costs increase with aging. Additionally, cost distribution pyramid demonstrated that hospital costs are greater in men compared to women before the age of 60 years old; however, it has been greater in women after the age of 60 years old than in men (Figure 1). 

**Table 1:** Comparison of the frequencies and hospital cost based on gender, nationality, age, LOS, and Season

**Variable**	**Type**	**Frequencies (%)**	**mean (SD) (million Rials)**	**95% CI**		***p*** **-value**
Gender	Male	6138 (%75.7)	21.73±5.14	20.44	23.01	<0.001^a^
	Female	1967 (%24.3)	14.71±4.34	12.79	16.63	
Nationality	Iranian	7412 (%91.4)	18.87±4.70	17.8	19.94	<0.001^ a^
	Foreigner	693 (%8.6)	32.38±7.10	27.08	37.68	
Age	0-15	1402 (%17.3)	12.96±2.94	11.46	14.46	<0.001^ b^
	16-30	3416 (%42.1)	19.53±4.47	17.86	21.2.	
	31-45	1783 (%22)	20.79±5.70	18.31	23.27	
	46-60	903 (%11.1)	21.28±4.73	18.34	24.21	
	61-75	426 (%5.3)	32.3±5.56	25.48	39.12	
	>80	142 (%1.8)	29.02±6.09	19.55	38.48	
	Unknown	33 (%0.4)	20.02±4.96	18.94	21.1	
Length of Stay (LOS)	Less than 1 day	4480 (%55.3)	4.44±1.20	4.3	4.58	<0.001^b^
	1-3 days	2405 (%29.7)	13.69±2.23	13.15	14.22	
	More than 3 days	1220 (%15)	89.74±14.25	84.08	95.39	
Season of the accident	Spring	1524 (%18.8)	19.51±4.57	17.21	21.81	0.06
	Summer	1704 (%21)	19.96±5.31	17.44	22.49	
	Autumn	1508 (%18.6)	22.99±5.86	20.02	25.95	
	Winter	1402 (17.3)	18.23±3.87	16.21	20.26	
	Unknown	1967 (%24.3)	-	-	-	

^a^ Independent-Sample T Test; ^b^ ANOVA

**Table 2:** Compare the frequencies and hospital cost based on site of accident, type of injury, type of vehicle and Patient’s final status

**Variable**	**Type**	**Frequencies (%)**	**mean (SD) (million Rials)**	**95% CI**		***p-*** **value**
Site of accident	Suburban highway	11 (%0.1)	86.29±9.02	25.68	146.91	<0.001^ b^
	Urban highway	12 (%0.1)	60.56±13.2	23.63	144.76	
	Suburban road	10 (%0.1)	69.63±7.90	13.11	126.16	
	Rural road	22 (%0.3)	33.14±4.89	11.46	54.83	
	Urban streets	8050 (%99.3)	19.77±4.92	18.7	20.85	
Type of injury	Injuries to the head	5010 (%61.8)	20.47±5.56	18.93	22.02	<0.001^ b^
	Injuries to the ankle and foot	267 (%3.3)	6.65±2.96	4.37	8.93	
	Injuries to the neck	391 (4.8%)	37.86±2.28	23.49	52.23	
	Injuries to the thorax	108 (%1.3)	13.03±7.53	7.9	18.16	
	Injuries to the abdomen, lower back, lumbar spine, pelvis and external genitals	265 (%3.3)	18.7±4.24	14.35	23.05	
	Injuries to the shoulder and upper arm	246 (%3.0)	16.21±3.67	12.51	19.92	
	Injuries to the elbow and forearm	267 (%3.3)	17.88±3.07	14.17	21.59	
	Injuries to the wrist, hand and fingers	206 (%2.5)	52.86±2.69	44.3	61.42	
	Injuries to the hip and thigh	247 (%3)	18.13±6.82	16.5	19.76	
	Injuries to the knee and lower leg	1098 (%13.5)	15.09±2.75	12.72	17.45	
Type of vehicle at the time of accident	Car	1783 (%22)	9.59±2.81	8.28	10.9	<0.001^ b^
	Motor cycle	5385 (%66.4)	23.64±5.50	22.17	25.11	
	Bicycle	128 (%1.6)	10.92±2.90	5.84	16.01	
	Passer-by	777 (%9.6)	20.3±4.82	16.91	23.7	
	Others	32 (%0.4)	23.07±4.76	5.87	40.26	
Patient’s final status	referral to other centers	51 (%0.6)	18.33±3.46	8.59	28.07	<0.001^ b^
	Full recovery	3739 (%46.1)	16.35±4.52	14.9	17.8	
	Relative recovery	3704 (%45.7)	22.57±4.99	20.96	24.18	
	Discharge with drug prescription	11 (%0.1)	18.37±2.56	1.11	35.63	
	Discharge against medical advice	442 (%5.5)	5.78±0.74	5.08	6.48	
	Escape	65 (%0.8)	54.23±11.8	24.85	83.62	
	Death	93 (%1.1)	111.44±10.8	89.03	133.86	

^a^ Independent-Sample T Test, ^b^ANOVA

## Discussion

In most countries, financing the treatment of injured people in traffic accidents is a separate fund that is paid from car insurance, public insurance or government fees [18, 19]. This is done in order to provide the necessary services as quickly as possible to RTI victims regardless of their financial statuses. Thus, given that the financial costs of these victims are secured from general sources, investigating RTI victims’ hospital costs and factors affecting them are of utmost importance in planning budgeting and allocating resources. 

Our results show that the most frequently used vehicle at the time of accident was motor cycle. Correspondingly, this is consistent with other studies [20-23]. Considering that motor cycle is the most frequently used vehicle in Yazd, most motor cyclists are teenagers and youth with no driving license. They have no financial capacity to buy a car, and these age groups observe safety rules to a lesser degree; therefore, they face traffic accidents more frequently. There was a significant difference between hospital costs and the type of vehicle used at the time of accident occurrence. Most costs pertained to motor cyclists and passerby. Given that passerby and motor cyclists enjoy the least physical safety and security, they sustain most injuries in accidents, leading to their increased treatment costs. Previous studies also mentioned passerby as a group of traffic accident victims [24, 25]. 

In the present study, %99.3 of the investigated accidents occurred in urban roads; however, there was a significant difference between hospital costs and location of accident, so that accidents occurring in suburban highways imposed higher costs on victims. This is consistent with the findings of other studies [20, 21, 26]. The greater number of traffic accidents in urban areas may be due to greater density of population, the use of motor vehicles, and the presence of passerby. Nevertheless, the high costs of RTI in suburban highways can be attributed to collisions with long vehicles at higher speeds as well as creating deeper injuries in passengers. 

Most accidents (%75.7) occurred to men, the costs of whom were 1.5 times greater than those of women. This is consistent with the results of previous studies [23, 27, 28]. Since men spend more hours out of home than women and women do not usually drive vehicles and motor cycles for long time, men are more likely to be exposed to RTAs. Of RTI patients, %17.3 were in the 0-15 years old age group and %42.1 of them were in the 16-30 years old age group. There was a significant correlation between age and hospital costs, in a way that the costs enhanced with ageing. In the study by Sing *et al*., most accidents occurred in the 20-40 years old age group [27]. The results of the studies conducted in China [29], India [23], and Vietnam [22] also confirmed our findings. Of note, this age group has greater potential for accidents as they are busier, go back and forth more frequently, and have a greater inclination for higher speeds. The mean length of stay (LOS) of accident victims was 1.94 days, and %55.3 of patients was hospitalized for less than 1 day. This rate was reported for all ambulatory and hospitalized patients. Thus, excluding the ambulatory victims, the mean LOS was 4.34 days and the patients’ hospital costs increased along with LOS increasing. The results of studies conducted in Belgium [30] and Iran [31] also reported a similar LOS for traumatized patients. Nevertheless, the patients’ LOS reported in this study is less than those reported in studies in Trinidad & Tobago [32], Kenya [33], Spain [34], and Greece [35], whereas it is greater than that reported in high-income countries [36]. 

The mean of total costs of patients was estimated as 20.02 million Rials. This rate is less than the amounts reported in high-income countries [33, 36], which may be attributed to low cost of healthcare services and lower tariff of services in Iran. Regarding the patient’s final status, %91.8 of the patients were discharged with full and partial recovery and only %1.1 of the victims died. Still, these patients had the highest costs due to expensive care for severe injuries. Accordingly, this is consistent with other studies [16, 36]. 

Regarding the type of injury, most injuries occurred in the cephalic region so that head injuries were observed in %61.8 of the victims. The costs of injuries of hand and fingers, neck, and head were higher than those of other parts of the body. These findings are consistent with the results of previous studies performed in Iran [37] and other countries [38-40]. In this regard, some studies have indicated that the use of helmets reduces mortality rate by %70 and diminishes severe damages by %40. However, the use of helmet is not very common among motor cyclists [1]. Consequently, since most accidents occur among motor cyclists who rarely use protective equipment, the injuries to head, neck, and hands are more common, which in turn require more vital and costly cares.

Our findings showed that hospital costs are higher for “men, the older people, dead victims, motor cyclists, LOS more than 3 days, accidents in the suburban highways, and head and neck traumas”. There was a significant correlation between these variables and hospital costs. These results may be useful in planning and designing some strategies for controlling RTAs and diminishing hospital costs of RTI victims. Nevertheless, taking media-based preventive measures for all age groups and teenagers at schools, in order to encourage the use of helmet by motor cyclists and spread the public culture of observing the rights of passersby may be fruitful. 

## Limitations

This study explored trauma information of both ambulatory and hospitalized patients (inpatients and outpatients). This point should also be taken into account in future studies. 
